# Supplementary material for: Stem Cells for Cancer Therapy: Translating the Uncertainties and Possibilities of Stem Cell Properties into Opportunities for Effective Cancer Therapy
Source: Int J Mol Sci. 2023 Jan 5;24(2):1012. doi: 10.3390/ijms24021012 (PMC9864033; doi:10.3390/ijms24021012)
Supplement: Supplementary file 1 [file ijms-24-01012-s001.zip › ijms-2117209-supplementary.pdf]

Review

Stem Cells for Cancer Therapy: Translating the Uncertainties and Possibilities of Stem Cell Properties into Opportunities for Effective Cancer Therapy

Ahmed Faris Aldoghachi [1], Zhi Xiong Chong [2], Swee Keong Yeap [3], Soon Keong Cheong [4], Wan Yong Ho [2] and Alan Han Kiat Ong [1,*]

[1] M. Kandiah Faculty of Medicine and Health Sciences, Universiti Tunku Abdul Rahman, Cheras 43000, Malaysia
[2] Division of Biomedical Sciences, School of Pharmacy, Faculty of Sciences and Engineering, University of Nottingham Malaysia, Semenyih 43500, Malaysia
[3] China-ASEAN College of Marine Sciences, Xiamen University Malaysia, Sepang 43900, Malaysia
[4] National Cancer Council (MAKNA), Kuala Lumpur 54000, Malaysia
* Correspondence: onghk@utar.edu.my

Table S1: Roles of circulating CSC-regulating miRNAs in modulating cancer cell stemness and its linkage to disease progression and prognosis (n=28).

Cancer type	miRNA	Targets	Study model (miRNA source)	Effects on cancer stemness	Effects on disease staging	Effects on cancer therapy	Effects on survival or relapse	Ref
Breast cancer	miR-130a-3p	↓ RAB5B	Clinical (Blood exosomes)	↓ Stemness	↓ Staging and metastases	NIA	NIA	[104]
Breast cancer	miR-155	↑ BMI1<br>↑ SLUG<br>↑ SNAIL<br>↑ SOX9<br>↑ EZH2	*In vitro* (Exosomes)	↑ Stemness	↑ Metastases	↑ Chemoresistance	NIA	[94]
Breast cancer	miR-93-3p<br>miR-105	↓ SFRP1	Clinical (Plasma)	↑ Stemness	↑ Staging and metastases	↑ Chemoresistance	↓ Survival	[103]
Breast cancer	miR-9-5p<br>miR-195-5p<br>miR-203a-3p	↓ ONECUT2	*In vitro* (Extra-cellular vesicles)	↑ Stemness	NIA	↑ Chemoresistance	NIA	[92]
Colorectal cancer	miR-17-5p	↓ SPOP<br>↑ PD-L1	*In vitro* (Exosomes)	↑ Stemness	NIA	NIA	NIA	[122]
Colorectal cancer	miR-19b	↓ FBXW7	*In vitro* (Exosomes)	↑ Stemness	NIA	↑ Radioresistance	NIA	[93]
Colorectal cancer	miR-146a-5p	↓ NUMB	*In vitro* (Exosomes)	↑ Stemness	NIA	NIA	NIA	[107]

Colorectal cancer	miR-375-3p	↓ B-catenin<br>↓ Vimentin<br>↓ ZEB1<br>↓ SNAIL	*In vitro* (Exosomes)	↓ Stemness	NIA	NIA	NIA	[105]
Colorectal cancer	miR-30a<br>miR-222	↓ MIA3	*In vitro* (Exosomes)	↑ Stemness	↑ Metastases	NIA	NIA	[108]
Gastric cancer	miR-500a-3p	↓ FBXW7	Clinical (Plasma) and *in vitro* (Exosomes)	↑ Stemness	↑ Staging	↑ Chemoresistance	↓ Survival	[90]
Glioblastoma	miR-30b-3p	↓ RHOB	Clinical (Serum) and *in vitro* (Extracellular vesicles)	↑ Stemness	↑ Grading	↑ Chemoresistance	↓ Survival	[111]
Glioblastoma	miR-504	↓ GRB10	*In vitro* (Extracellular vesicles)	↓ Stemness	↓ Grading	NIA	↑ Survival	[98]
Glioblastoma	miR-603	↓ IGF1<br>↓ IGF1R	*In vitro* (Extracellular vesicles)	↓ Stemness	NIA	↓ Radioresistance	↑ Survival	[100]
Glioblastoma	miR-9-5p miR-124-3p	↓ VIM	*In vitro* (Extracellular vesicles)	↑ Stemness	NIA	NIA	↓ Survival	[123]
Glioblastoma	miR-16-5p<br>miR-23a-3p<br>miR-144-3p<br>miR-155-5p<br>miR-320e	↓ PTEN	*In vitro* (Extracellular vesicles)	↑ Stemness	↑ Metastases	↑ Radioresistance	↓ Survival	[91]

	miR-363-3p<br>miR-495-3p<br>miR-520f-3p							
Liver cancer	miR-1246	↓ AXIN2<br>↓ GSK-3β	Clinical (Plasma)	↑ Stemness	↑ Metastases	↑ Chemoresistance	↓ Survival	[96]
Liver cancer	miR-125a<br>miR-125b	↓ CD90	*In vitro* (Exosomes)	↓ Stemness	↓ Metastases	NIA	↓ Survival	[95]
Oral squamous cell carcinoma	miR-21<br>miR-34	NIA	*In vitro* (Exosomes)	↓ Stemness	NIA	NIA	NIA	[116]
Pancreas	miR-210	↑ mTOR	*In vitro* (Exosomes)	↑ Stemness	↑ Invasion	↑ Chemoresistance	NIA	[101]
Lung cancer	miR-210-3p	↓ FGFRL1	*In vitro* (Exosomes)	↑ Stemness	↑ Metastases	NIA	NIA	[97]
Clear cell renal cell carcinoma	miR-19b-3p	↓ PTEN	Clinical (Blood) and *in vitro* (Exosomes)	↑ Stemness	↑ Staging and metastases	NIA	NIA	[113]
Prostate cancer	miR-139-5p<br>miR-148a-3p<br>miR-183-5p<br>miR-1307-3p<br>miR-1307-5p<br>miR-7641	↑ miR-139-5p MMP-2, MMP-9, MMP-13, RANKL	*In vitro* (Exosomes)	↑ Stemness	↑ Metastases	NIA	NIA	[99]

Multiple myeloma	miR-1305	↓MDM2<br>↓ IGF1<br>↓ FGF2	Clinical (blood) and *in vitro* (Exosomes)	↓	Stemness	NIA	NIA	↑ Exosomal miR-1305 ↓ cellular miR-1305 and led to ↓ survival	[117]
Acute myeloid leukemia	miR-9	↓ HES1	Clinical (Blood)	↑	Stemness	NIA	NIA	NIA	[118]
Acute myeloid leukemia	miR-34c-5p	↓ RAB27B	Clinical (blood) and *in vitro* (Exosomes)	↓	Stemness	NIA	NIA	↑Survival	[119]
Myelodysplastic syndrome and leukemia	miR-22	↓ TET2	Clinical (Blood)	↑	Stemness	NIA	NIA	↓ Survival	[120]

Abbreviation: NIA: no information available.
